# Medical Ministry

**Published:** 1883-08-20

**Authors:** 


### *MEDICAL MINISTRY.*

Perhaps there is no word of more significance, and more suggestive of responsibilities and imperative duties, than the word ministry. To minister is to attend, to serve, to supply things needful, to give relief. What an important work is here suggested to the mind, full of noble, faithful, and self-sacrificial performance of the highest obligations resting upon the ministering actor in the work.

To minister, in its truest and best significance, is emphatically a labor of love. It must be an unselfish work—a work of time, of zeal and devotion; an intelligent work, comprehending with clearness and precision its duties and action, and finally, it should be a work in which the whole heart, mind and energies are engaged so that successful results may follow its performance. The lawyer ministers to his clients; the politician to his constituents; the merchant to his patrons, and the instructor to his pupils; but, with the exception of the embassadors of the Gospel and the mother whose ministrations of love are ceaseless in her unwearied devotion to her child, on no class of persons do these obligations rest as fully and imperatively as upon the minister of medicine.

But though the duties and labors are so great in this work, it has its compensations nobler and better than those of any other profession of an earthly character, It has been said of the philanthropist that,

> "The drying up of a single tear has more
> Of honest fame than shedding seas of gore."

But who can estimate the worth of the true physician in his ministry of love and humanity to these frail bodies, the harmonious action of which is so easily disturbed by disease, and which are targets at all times for the shafts of man's last dread enemy? Let every physical pain he has mitigated, every rose of health he planted upon the pallid cheek, and every fellow-creature he has restored from an untimely grave, answer the question.

When a physician hears a patient ask, "Doctor, cannot you do something for me?" or a suffering woman cry out in her agony and fear, "Doctor, do help me!" a pure and great joy thrills his heart when he feels that he can relieve the pain of that helpless woman, and turn her appealing cries of distress into smiles of joy and glad words of grateful thanks to her preserver. Yes, these frequent oases are full of strength and refreshment to the ministering physician in his weary and often thankless labor; they are sweet compensations for his toil and devotion, and pure incentives to a still higher and fuller performance of duty.

The true ministry of medicine is Christ-like in its life and character. I say this with all reverence, and it is the highest compliment I can pay the profession, whether regarded in a social, intellectual, or professional light. Like the great healing Exemplar, the true physician goes about doing good. Curing disease, not indeed by miracles, but by a thorough and intelligent knowledge of his work, and a consciousness of his power, and also, by his moral example before those with whom he comes in contact, socially or professionally.

The minister of medicine must also go whenever and wherever he is called upon to give relief to a sufferer.

There is no record where the Savior of mankind ever refused to attend a call upon His mercy, or a claim upon His power; and the physician who fails to answer a cry for help from his fellow-man where it is possible for him to respond and give relief, is false to one of the highest obligations of his ministerial work. For here, Christ-like also, he never wearies in well-doing. Love for the brotherhood of man in its periods of pain and distress, and a profound sense of the greatness of his vocation, are continual incentives to action, and give him an energy that never flags, while he feels a just pride and pure pleasure in the daily and constant exercise of his skill and capacity as a minister to suffering humanity.

Even fear of the dire pestilence does not deter him from the discharge of his conscientious duties, but putting aside all selfish and personal considerations, he offers himself, if need be, a holocaust upon the altar of man's love to his fellow-man, and adds another name to the long list of earth's dead and uncrowned heroes.

T. S. P.

Blue Ridge Springs, Va., Aug. 15, 1883.

[Concluded next month.]

